# Comparative differences in metabolic, mental health and perinatal outcomes of women with gestational diabetes in Ghana and Switzerland: the G-MUM study

**DOI:** 10.1186/s12884-025-07577-1

**Published:** 2025-04-15

**Authors:** Dan Yedu Quansah, Kelvin Yeboah, Floriane Schweitzer, Sandra Yedu Quansah, Evans Kofi Agbeno, Antje Horsch, Katrien Benhalima, A. Kofi Amegah, Jardena J. Puder

**Affiliations:** 1 Obstetric service, Department Woman-Mother-Child, Lausanne University Hospital, Rue du Bugnon 21, Lausanne, CH-1011 Switzerland; 2 Public Health Research Group, Department of Biomedical Sciences, School of Allied Health Sciences, University of Cape Coast, Cape Coast, Ghana; 3 Department of Education and Psychology, University of Cape Coast, Cape Coast, Ghana; 4 Department of Obstetrics and Gynecology, School of Medical Sciences, University of Cape Coast, Cape Coast, Ghana; 5 Institute of Higher Education and Research in Healthcare (IUFRS), University of Lausanne, Lausanne, Switzerland; 6 Neonatology service, Department Woman-Mother-Child, Lausanne University Hospital, Lausanne, Switzerland; 7 Department of Endocrinology, UZ Gasthuisberg, KU Leuven, Leuven, Belgium

**Keywords:** Metabolic, Mental health, gestational diabetes, Eating behaviour, Ghana, Switzerland, Obstetric, Neonatal

## Abstract

**Background:**

Gestational diabetes mellitus (GDM) prevalence (9–15%) is similar in Ghana and Switzerland, despite differences in sociodemographic characteristics, lifestyle, and healthcare systems. Contrary to Switzerland, data on the metabolic and mental health outcomes of women with GDM in Ghana is lacking. We compared the metabolic, mental health, and perinatal outcomes of GDM during pregnancy in Ghana and Switzerland.

**Methods:**

This prospective observational study included 170 women with GDM from two cohorts (*n* = 88 in Switzerland, *n* = 82 in Ghana) who were followed-up until delivery. Primary outcomes were metabolic [HbA1c, gestational weight gain (GWG)], well-being (WHO-5), and depression symptoms (EPDS) during pregnancy. Secondary outcomes were obstetric and neonatal outcomes. Differences in metabolic, mental health and perinatal outcomes were tested using ANOVA, Chi-square test or ANCOVA when appropriate. For the perinatal outcomes, we adjusted for metabolic confounders.

**Results:**

Compared to the Swiss cohort (SC), the Ghana cohort (GC) had a higher pre-pregnancy weight (79.8 ± 18.0 vs. 71.8 ± 15.3 kg, *p* = 0.003) and BMI (30.8 ± 6.6 vs. 26.5 ± 4.9 kg/m^2^, *p* < 0.001), lower total GWG (6.2 ± 5.4 vs. 11.3 ± 5.8 kg, *p* < 0.001), but similar BMI-based excessive weight gain, higher fasting glucose (7.1 ± 2.4 vs. 5.1 ± 0.7 mmol/l) and HbA1c at GDM diagnosis (5.6 ± 1.0 vs. 5.3 ± 0.3%; both *p* ≤ 0.019), higher prevalence of previously undiagnosed pre-existing diabetes before 15-weeks gestational age (35.4% vs. 9.4%) and of metformin-treated women (84% vs. 1.1%, both *p* ≤ 0.001). The GC had higher well-being scores (74.4 ± 17.6 vs. 59.8 ± 18.3, *p* < 0.001), but similar symptoms of depression scores. In the GC, LGA (30% vs. 17%), and NICU hospitalizations (42% vs. 15%) were higher (all *p* ≤ 0.044) independent of HbA1c and pre-pregnancy BMI.

**Conclusion:**

Women in the GC had worse metabolic outcomes but improved mental health during pregnancy. In this context, LGA and neonatal hospitalisations were higher, independent of HbA1c and pre-pregnancy BMI. Our results suggest the need for specialized GDM clinics in Ghana to improve metabolic and perinatal outcomes in women with GDM.

## Introduction

Gestational diabetes mellitus (GDM) is any degree of glucose intolerance or hyperglycaemia with first recognition during pregnancy [1]. GDM is one of the most common pregnancy complications affecting around 3–20% of pregnant women globally [2]. About 11% of all pregnancies in Switzerland [3] and 9–15% of women attending antenatal clinics in Ghana develop GDM [4, 5].

GDM of any severity increases the risk of fetal and maternal complications [6–11]. Long-term complications of GDM such as diabetes and cardiovascular diseases also depend on pre-pregnancy weight and on weight retention, both of which are closely linked to gestational weight gain (GWG). Up to 60–70% of women with GDM are overweight/obese before pregnancy [12]. A higher maternal pre-pregnancy weight (especially BMI ≥ 25 kg/m^2^) and excessive GWG in women with GDM is highly predictive of adverse maternal and neonatal outcomes [13].

Higher stress exposure, perceived stress during pregnancy [14], and higher symptoms of depression early in pregnancy increase the risk of GDM [15]. Compared to women without GDM, women with GDM are 2–4 times more likely to develop symptoms of depression during pregnancy [15]. Depression symptoms are associated with poor eating behavior and decreased physical activity, as well as excess weight gain and subsequent postpartum weight retention [16]. There is a bi-directional relationship between poor eating behaviors and symptoms of depression in women with GDM [17].

To date, studies conducted in women with GDM in Ghana only focused on the different diagnostic criteria, GDM prevalence, its risk factors [4, 5], and on obstetric outcomes [18]. No study has investigated mental health outcomes, and data on metabolic outcomes of GDM during pregnancy are limited. A recent study in Ghana found that excess weight gain and high caloric food intake were significant risk factors for GDM [5]. Despite the differences in sociodemographic characteristics, lifestyle, and healthcare systems in Switzerland and Ghana, the prevalence of GDM is similar. For example, whereas Switzerland has a well-defined routine GDM diagnosis, follow-up, and treatment and management guidelines, this was not the case in Ghana even though obesity, a significant risk factor of GDM continues to increase in Ghana. Although the current prevalence is still unknown for 2023 or 2024, obesity was predicted in 2008 to increase by 15% among Ghanaian women of reproductive age by 2023 [19]. Therefore, to tailor interventions to the cultural context of Ghana, there is a need to understand and evaluate the metabolic and mental health outcomes of GDM during pregnancy. The overall goal of the Gestational Diabetes Mothers (G-MUM) study was to investigate and compare the maternal metabolic and mental health outcomes, as well as the obstetric and neonatal outcomes of women with GDM in Ghana and Switzerland.

## Methods

### Study design and patient population

This observational study compared the metabolic and mental health outcomes of two cohorts of women with GDM in Switzerland and Ghana. We followed women until delivery between 1 March 2022 and 31 August 2022 in both cohorts. In Switzerland, we recruited women from the diabetes and pregnancy clinic at the Lausanne University Hospital (CHUV). The CHUV is the tertiary hospital in the province of Vaud where both women from the city and neighboring towns (urban) and villages (rural) are referred. In Ghana, we recruited women attending antenatal clinic at the maternity unit of two study centers: the University of Cape Coast Hospital and the Cape Coast Teaching Hospital. Cape Coast metropolis is the capital city of the central region of Ghana. Cape Coast teaching hospital is the referral hospital of the region where women from both the city (urban) and rural areas are referred. Details of the Swiss cohort (SC) have been previously described [20–23]. The Human Research Ethics Committee of the Canton de Vaud (326/15) and the University of Cape Coast Institutional Review Board (UCCIRB/EXT/2021/39) approved the study protocols for the SC and the Ghana cohort (GC) respectively.

Both cohorts included women with GDM aged ≥ 18 years. We excluded women on strict bed rest, with pre-existing diabetes or known severe mental disorder, and those on insulin treatment before diagnosis. In total, 170 women (*n* = 88 in the SC and *n* = 82 in the GC) were included. The reporting of this study is consistent with strengthening the reporting of observational studies in epidemiology (STROBE) guideline.

### GDM diagnosis, treatment, and patient follow-up

In both cohorts, women were diagnosed with GDM if at least one of the following criteria was fulfilled: fasting glucose ≥ 5.1 mmol/L, 1 h ≥ 10.0 mmol/L, 2 h ≥ 8.5 mmol/L during a 75-g oral glucose tolerance test (OGTT) according to the International Association of Diabetic Pregnancy Study Groups (IADPSG) criteria [24]. In addition, GDM screening was performed in both the SC and the GC according to the usual clinical procedure in both countries. In the SC, all women were tested for GDM at 24–32 weeks gestational age (GA) using either directly a 75 g OGTT or they underwent a fasting glucose test and women with fasting glucose values ≥ 4.4 mmol/l and < 5.1 mmol/l had a 75 g OGTT to diagnose GDM [1]. In the GC, all women underwent a fasting glucose test at the first antenatal clinic (ANC) booking, usually in the first or the early second trimester, and those with fasting glucose values ≥ 4.4 mmol/l and < 5.1 mmol/l had a 75 g OGTT to diagnose GDM [25]; as a result, women were diagnosed between 18 and 32 weeks GA. If results were negative, they were not repeated later.

Women in the SC were routinely followed-up clinically according to the current international guidelines [26, 27]. They had continuous regular face-to-face appointments every 1–3 weeks with a medical doctor, a diabetes-specialist nurse and/or a dietician after the GDM diagnosis. During these visits, women received information on GDM, specific recommendations regarding lifestyle changes and gestational weight gain (GWG) based on the 2009 recommendations of the National Academy of Medicine (NAM; previously Institute of Medicine) [28]. Physical activity was encouraged and counselling with a physiotherapist and/or participation in GDM physical activity groups was proposed. Treatment with insulin or rarely with metformin, was introduced when glucose values remained above targets between two or more times during a 1-2-week period despite lifestyle changes, in line with Swiss guidelines [29]. Women in the GC had regular face-to-face antenatal care appointments every 1–3 weeks with the doctor and/or with a dietician after diagnosis. They received information on lifestyle changes and on weight gain during pregnancy based on existing antenatal care guidelines in Ghana. Women with a need for glucose-lowering medication treatment were mostly treated with metformin and not with insulin. The decision for medication treatment was made when glucose values remained above targets between two or more times during a 1–2 week of using glucometers for those who can afford (not covered by insurance) or every 1–3 days visits to the antenatal clinic to check glucose levels for two weeks.

### Outcomes

Outcomes were assessed at diagnosis, at the end of pregnancy, and at birth. Primary outcomes included metabolic (GWG, rate of weight gain per week) and lifestyle/mental health measures (symptoms of depression, well-being, intuitive eating) outcomes. Obstetric and neonatal outcomes were secondary outcomes.

#### Sociodemographic variables

Data on maternal socio-demographic characteristics including age, nationality/ethnic origin, and educational level were collected at inclusion (the first GDM visit). We extracted information on previous history of GDM, family history of diabetes, gravida, parity, and social support during pregnancy (living with partner or with support, yes/no) from participants’ medical charts in the SC and with a structured questionnaire in the GC. Pre-pregnancy weight was extracted from participants’ medical charts or, if missing, was self-reported (entirely self-reported in the GC).

#### Metabolic health outcomes

We measured height and weight at inclusion (18–32 weeks GA) and last GDM visit, to the nearest 0.1 cm and 0.1 kg, respectively, with electronic scales (Seca^®^ Model 7017021094) in both cohorts. BMI was expressed as a ratio of weight in kilograms to the square of height in meters (kg/m^2^). Total GWG was defined as the difference in weight at the end of pregnancy and pre-pregnancy weight whereas GWG since the first GDM visit was defined as the difference in weight at the first visit and weight at the end of pregnancy. We calculated the rate of weight gain per week during pregnancy. Excessive GWG was calculated according to NAM 2009 GWG recommendations based on pre-pregnancy BMI [28]. Blood pressure was measured with a digital blood pressure monitor (OMRON^®^ HEM-907) at inclusion and at the end of pregnancy. Data on the need for glucose-lowering medical treatment during pregnancy (use of insulin and/or metformin; yes/no) were extracted from maternal medical records. Fasting, 1 h and 2 h glucose was measured during the GDM diagnosis. HbA1c was assessed at the first GDM visit using a chemical photometric method (conjugation with boronate; Afinion^®^) [30]. We diagnosed diabetes in pregnancy for women tested and diagnosed before 15 weeks GA according to the American Diabetes Association (ADA) criteria (FPG ≥ 7.0 mmol/L, 2-hour glucose ≥ 11.1 mmol/L or HbA1c ≥ 6.5%) [1].

#### Mental health outcomes

Maternal mental health outcomes included symptoms of depression, well-being, intuitive eating, and timing of food intake during pregnancy. We used the Edinburgh Postnatal Depression Scale (EPDS) (validated 10-item questionnaire) to measure symptoms of depression [31]. The EPDS has possible scores of 0–30 points; a higher total score indicates more severe depressive symptoms. Maternal well-being was assessed with the World Health Organization (WHO-Five) Well-Being Index [32], a validated 5-item self-report questionnaire assessed on a 5-point Likert scale ranging from 0 ‘*at no time*’ to 5 ‘*all of the time*’. Total score of the five-items is then multiplied by four [4] to obtain a final score. Possible scores range from 0 to 100, and higher scores reflect higher well-being status. We assessed intuitive eating with two out of the three subscales of French Intuitive Eating Scale-2 (IES-2): the “eating for physical rather than emotional reasons” (EPR, 8 items) and the “reliance on hunger and satiety cues” (RHSC, 6 items) subscales [33]. We removed the unconditional permission to eat (UPE) subscale as women involved in the study had in general at least one visit with a registered dietician before responding to the IES-2. Thus, discussions during the diet counselling could significantly influence participant responses to the UPE subscale questions such as “I try to avoid certain foods high in fat, carbohydrates, or calories”. Scores of IES-2 range between 1 and 5 for each subscale. A higher score of the EPR subscale reflects eating as an answer to hunger and a lower score means eating to cope with emotional distress, whereas a higher score of the RHSC subscale signifies trust in internal cues, and a lower score reflects less ability to regulate food intake. Timing of food intake behaviour was assessed with the 7-item timing of food intake questionnaire [34]. This questionnaire assesses the time of food intake in the past seven days. This includes the time of first meal or food intake, time of last main mean, time of last food intake, time of first and last drink intake, number of food intakes (eaten) during a day as well as the number of breakfast intakes in a week.

#### Obstetric and neonatal outcomes

Obstetric outcomes included GA at birth (weeks), sex, preterm birth (< 37 GA) and caesarean section. Neonatal adverse outcomes included macrosomia (birthweight ≥ 4000 g), large-for-gestational-age (LGA) and small-for-gestational-age (SGA) infants. LGA and SGA were defined as sex-and GA-specific birth weight > 90th and < 10th centile, respectively, according to the International Fetal and Newborn Growth Consortium for the 21st Century (INTERGROWTH-21st) guidelines [35]. Other neonatal outcome measures were birthweight, birth length, head circumference and their z-scores, the latter calculated according to INTERGROWTH-21 guidelines [35]. Additional outcomes were Apgar scores at 1 and 5 min, and prevalence of neonatal intensive care unit (NICU) hospitalization. Data on neonatal complications including hypoglycemia and respiratory distress were mostly missing in the GC as it was not available in the medical charts and thus not included in the analysis.

### Statistical analysis

We performed all statistical analyses with Stata/SE 15.1 (StataCorp LLC, TX, USA) [36]. Demographic and other descriptive variables were presented as means (± standard deviation) or percentages (%) where appropriate. The differences in maternal socio-demographic and clinical characteristics between both cohorts were investigated using ANOVA or Chi-square test where appropriate. All outcome variables (weight, GWG, HbA1c, EPDS score, well-being score, EPR and RHSC, obstetric and neonatal variables) were normally distributed. We compared the differences in women who had excessive GWG as well as the differences in NAM (previously IOM) weight gain recommendation (Adhere, Below or Above) in both cohorts. We determined and compared the differences in maternal metabolic and mental health outcomes during pregnancy as well as the differences in obstetric and neonatal outcomes. Regarding the differences in obstetric and neonatal outcomes in both cohorts, we adjusted for the metabolic confounders HbA1c at GDM diagnosis and pre-pregnancy BMI using an analysis of covariance (ANCOVA). We thus presented only adjusted results. All statistical significances were two sided and accepted at *p* < 0.05.

## Results

The G-Mum study included 170 women from two cohorts (Table 1). 52% (*n* = 88) of the women were in the SC and 48% (*n* = 82) were in the GC. Of these, 45% (*n* = 40/88) and 85% (*n* = 70/82) of women in the SC and GC, respectively, had obstetric and neonatal outcomes data at the time of this analysis. The mean age, pre-pregnancy weight and pre-pregnancy BMI were 34.6 ± 4.9 years, 75.3 ± 16.9 kg and 28.4 ± 6.1 kg/m^2^ respectively. Compared to the SC, the GC were diagnosed with GDM at a mean of 9.1 weeks earlier (18.7 ± 8.2 vs. 27.8 ± 3.4 weeks), had a higher pre-pregnancy BMI but lower GWG up to the baseline visit (all *p* ≤ 0.005). Around two-thirds of the SC had a family history of diabetes compared to around one third in the GC (60% vs. 36%, *p* = 0.004).

**Table 1:** Baseline socio-demographic and clinical characteristics in women diagnosed with gestational diabetes in Ghana and Switzerland

Variable	Switzerland (*n* = 88)	Ghana (*n* = 82)	*P*-value
	*n*, %	*n*, %	
Age (year), *mean ± SD*	34.7 ± 4.4	34.5 ± 5.5	0.766
GA at the first GDM visit (weeks) *mean ± SD*	27.8 ± 3.4	18.7 ± 8.2	**< 0.001**
Pre-pregnancy weight (kg) *mean ± SD*	71.8 ± 15.3	79.8 ± 18.0	**0.003**
Pre-pregnancy BMI (kg/m^2^) *mean ± SD*	26.5 ± 4.9	30.8 ± 6.6	**< 0.001**
BMI at the first GDM visit (kg/m^2^) *mean ± SD*	30.3 ± 4.8	33.0 ± 6.6	**0.005**
GWG up to baseline visit (kg) *mean ± SD*	10.1 ± 5.3	4.5 ± 4.8	**< 0.001**
Ethnicity/Nationality			
Africa	11 (12.5)	82 (100)	**< 0.001**
Europe and North America	67 (76.14)	0	
Asia and Oceania	10 (11.36)	0	
Education level^1^			
No formal education	3 (6.0)	4 (4.88)	**< 0.001**
Compulsory school incomplete*	1 (2.0)	15 (18.29)	
High school	15 (30.0)	16 (19.51)	
General and vocational education	16 (32.0)	0	
University	15 (30.0)	47 (57.32)	
Glucose-lowering treatment in pregnancy			
None	63 (71.59)	7 (8.54)	**< 0.001**
Insulin	24 (27.27)	6 (7.32)	
Metformin	1 (1.14)	69 (84.15)	
Parity			
0	43 (49.43)	26 (31.71)	**0.033**
1	27 (31.03)	24 (29.27)	
2	11 (12.64)	21 (25.61)	
≥3	6 (6.9)	11 (13.41)	
Gravida			
1	32 (36.78)	17 (20.73)	**0.014**
2	21 (24.14)	15 (18.29)	
≥3	34 (39.08)	50 (60.98)	
GDM in previous pregnancy			
Yes	7 (13.46)	20 (24.39)	0.184
Family history of diabetes^2^			
Yes	48 (60.0)	30 (36.59)	**0.004**
Social support during pregnancy^3^			
Yes	83 (94.32)	81 (98.78)	0.402

GA denotes gestational age; GDM denotes gestational diabetes mellitus; SD denotes standard deviation; BMI denotes body mass index, GWG denotes gestational weight gain

^1^38 participants in the Swiss cohort have missing data on education

^2^Family history of diabetes consists of those with first-degree relationship of the participant (e.g., mother, father, brother, sister, daughter, son)

^3^Yes denotes women who lives with partner or live alone with family support

*Compulsory school in Ghana is 9 years and that of Switzerland is 11 years

All values are expressed as n, % or mean (and standard deviation). *P* values derived from Chi-square test was used for categorical variables and ANOVA for continuous variables. Bold *p* values are significant (*p* < 0.05)

### Maternal metabolic health outcomes during pregnancy

**Fig. 1:**
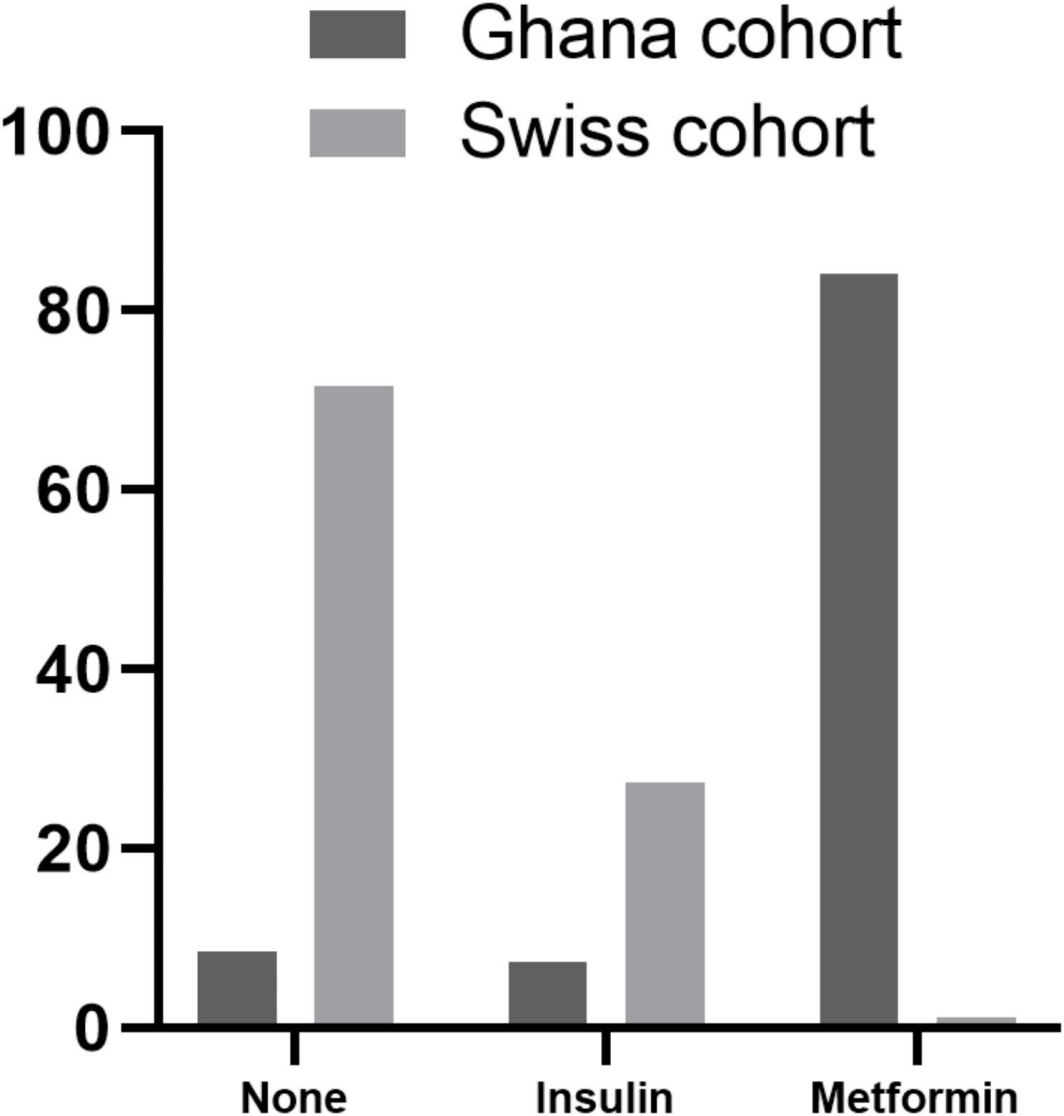
Glucose-lowering medication treatment during pregnancy in both cohorts. Compared to the Swiss cohort, 84% of women in the Ghana cohort received metformin compared to only 1% of the Swiss chort. On the other hand, 27% of the Swiss cohort received insulin compared to 7% of the Ghana cohort

Table 2 describes maternal metabolic health outcomes during pregnancy in both cohorts. There were no differences in weight at the first GDM visit or at the end of pregnancy between the two cohorts. However, the GC had significantly lower total GWG (6.2 ± 5.4 vs. 11.3 ± 5.8 kg) and lower weekly weight gain compared to the SC during the entire pregnancy (0.16 ± 0.1 vs. 0.30 ± 0.2 kg, both *p* < 0.001), while GWG since the first GDM visit was higher in the GC (2.1 ± 4.1 vs. 1.5 ± 2.9 kg, *p* = 0.039). Importantly, the weekly rate of GWG since GDM diagnosis was similar in both groups (0.14 ± 0.28 vs. 0.10 ± 0.35 kg). Excessive GWG for the entire pregnancy did not differ between both groups (17% vs. 13%, *p* = 0.531). Regarding glycemic parameters, fasting glucose, 2 h glucose, and HbA1c at GDM diagnosis were significantly higher in the GC (both *p* ≤ 0.019). Additionally the GC had a 3.7 times higher prevalence of diabetes diagnosed before 15 weeks GA in pregnancy (35.4% vs. 9.41%, *p* < 0.001) based on ADA criteria [1]. Figure 1 shows the differences in the use of glucose-lowering medication treatment during pregnant in both cohorts. The majority of women in the SC (72%) received no glucose-lowering medication treatment during pregnancy, whereas 84% of the women in the GC received metformin (*p* < 0.001). Although diastolic blood pressure at the first GDM visit was similar, women in the SC had a higher systolic blood pressure visit compared to the GC (117.2 ± 14.7 vs. 110.4 ± 15.0, *p* = 0.004). These values were similar at the end of pregnancy.

**Table 2:** Differences in maternal metabolic health outcomes during pregnancy in women diagnosed with gestational diabetes in Ghana and Switzerland

Variable	Switzerland (*n* = 88)	Ghana (*n* = 82)	*P*-value
	Mean ± SD	Mean ± SD	
Weight at the first GDM visit (kg)	82.3 ± 14.9	85.2 ± 17.5	0.265
Weight at the end of pregnancy (kg)	83.6 ± 15.5	87.3 ± 15.8	0.238
GWG since first GDM visit (kg)	1.5 ± 2.9	2.1 ± 4.1	**0.039**
Rate of GWG since GDM diagnosis (kg)	0.14 ± 0.28	0.10 ± 0.35	0.664
Total GWG during pregnancy (kg)	11.3 ± 5.8	6.2 ± 5.4	**< 0.001**
Rate of weight gain per week during pregnancy (kg)	0.30 ± 0.2	0.16 ± 0.1	**< 0.001**
Excessive GWG (kg) (n, %)			
Yes	15 (17.05)	11 (13.41)	0.531
No	73 (82.95)	71 (86.59)	
IOM recommendation for total GWG (kg) (n, %)			
Met recommendation	52 (59.09)	43 (52.44)	0.571
Below recommendation	16 (18.18)	20 (24.39)	
Above recommendation	20 (22.73)	19 (23.17)	
Fasting glucose at GDM diagnosis (mmol/l)	5.1 ± 0.75	7.1 ± 2.4	**< 0.001**
2 h glucose at GDM diagnosis (mmol/l)	8.1 ± 1.8	9.1 ± 2.6	**0.019**
HbA1c at the first GDM visit (%)	5.3 ± 0.3	5.6 ± 1.0	**0.004**
Suspicion of diabetes before 15 weeks GA, yes (n, %)^1^	8 (9.41)	29 (35.36)	**< 0.001**
SBP at the first GDM visit (mmHg)	117.2 ± 14.7	110.4 ± 15.0	**0.004**
DBP at the first GDM visit (mmHg)	72.7 ± 13.2	71.4 ± 11.0	0.485
SBP at the end of pregnancy (mmHg)	116.5 ± 10.2	116.7 ± 16.8	0.937
DBP at the end of pregnancy (mmHg)	75.6 ± 9.1	74.9 ± 11.4	0.751

GDM denotes gestational diabetes mellitus; SD denotes standard deviation; BMI denotes body mass index, GWG denotes gestational weight gain; IOM denotes Institute of Medicine; HbA1c denotes glycated hemoglobin; SBP denotes systolic blood pressure; DBP denotes diastolic blood pressure; GA denotes gestational age

^1^Suspicion of diabetes during pregnancy was diagnosed at less than 15 weeks gestational age according to ADA criteria (FPG ≥ 7.0 mmol/L, 2-hour glucose ≥ 11.1 mmol/L or HbA1c ≥ 6.5%)

Data is presented as mean ± standard deviation unless otherwise stated

*P* values derived from Chi-square test was used for categorical variables and ANOVA for continuous variables. Bold *p* values are significant (*p* < 0.05)

### Maternal mental health, intuitive eating behavior and timing of food intake during pregnancy

Table 3 shows the differences in mental health outcomes between both cohorts. The GC had higher well-being scores compared to the SC (74.4 ± 17.6 vs. 59.8 ± 18.3, *p* < 0.001) independent of HbA1c at GDM diagnosis and pre-pregnancy BMI. In analogy, the SC had slightly increased depressive symptoms during pregnancy, but this was not significantly different between both groups. Regarding intuitive eating behavior, the GC had increased “eating for physical rather than emotional reasons” (EPR) score (4.5 ± 0.5 vs. 3.6 ± 0.7, *p* < 0.001), whereas the SC had “higher reliance on hunger and satiety cues” (RHSC) scores (3.5 ± 0.7 vs. 3.1 ± 1.0, *p* = 0.007) (Table 3) even after adjusting for HbA1c and pre-pregnancy BMI. We found significant differences in the timing of food intake behavior between both cohorts. The times of first meal and last meal intakes were earlier in the GC (both *p* ≤ 0.011). When we compared the number of breakfast intakes per week in both groups, the GC had a higher number of breakfast intake (7.0 ± 2.0 vs. 5.1 ± 2.4, *p* < 0.001).

**Table 3:** Adjusted differences in maternal mental health, intuitive eating behavior, and timing of food intake during pregnancy in women diagnosed with gestational diabetes in Ghana and Switzerland

Variable	Switzerland (*n* = 88)	Ghana (*n* = 82)	*P*-value#	*P*-value*
	Mean ± SD	Mean ± SD		
**Mental health**				
Total depression symptoms score (EPDS)^1^	7.48 ± 4.69	6.92 ± 4.8	0.447	0.062
Symptoms of depression (n, %)				
Subclinical symptoms (EPDS < 11)	69 (77.91)	67 (81.71)	0.570	0.085
Probable diagnosis (EPDS ≥ 11)	19 (22.09)	15 (18.29)		
Total well-being score (WHO-5)^2^	59.85 ± 18.36	74.4 ± 17.6	**< 0.001**	**0.005**
**Intuitive eating behavior**				
EPR subscale^3^	3.67 ± 0.74	4.53 ± 0.59	**< 0.001**	**< 0.001**
RHSC subscale^4^	3.52 ± 0.75	3.15 ± 1.0	**0.007**	**0.015**
**Timing of food intake behavior**				
Time of first meal (hour)	8.55 ± 2.24	8.10 ± 2.11	**0.011**	**< 0.001**
Time of last main meal (hour)	19.20 ± 2.23	17.43 ± 1.27	**< 0.001**	**0.038**
Time of last food intake (hour)	19.5 ± 3.21	19.09 ± 3.55	0.285	0.351
Number of food intake in a day	3.6 ± 1.94	3.6 ± 1.21	0.907	0.117
Number of breakfast intakes in a week	5.16 ± 2.45	7.06 ± 1.95	**< 0.001**	**0.002**

^1^EPDS denotes Edinburg postnatal depression scale (higher total score indicates more severe depressive symptoms)

^2^The World Health Organization-Five Well-Being Index (WHO-5) (higher scores reflect higher well-being status and lower scores indicates lower well-being)

^3^EPR denotes eating for physical rather than emotions subscale of the intuitive eating scale-2 (IES-2) (higher score reflects eating as an answer to hunger and a lower score means eating to cope with emotional distress)

^4^RHSC denotes reliance on hunger and satiety cues subscale of the intuitive eating scale-2 (IES-2) (higher score signifies trust in internal cues, and a lower score reflects less ability to regulate food intake)

All values are expressed as mean and standard deviation unless otherwise stated

^#^Unadjsuted *p* values

* *P* values were adjusted for HbA1c at GDM diagnosis and for pre-pregnancy BMI

*P* values derived from Chi-square test was used for categorical variables and ANOVA (ANCOVA for adjusted results) for continuous variables. Bold *p* values are significant (*p* < 0.05)

### Obstetric and neonatal outcomes

**Fig. 2:**
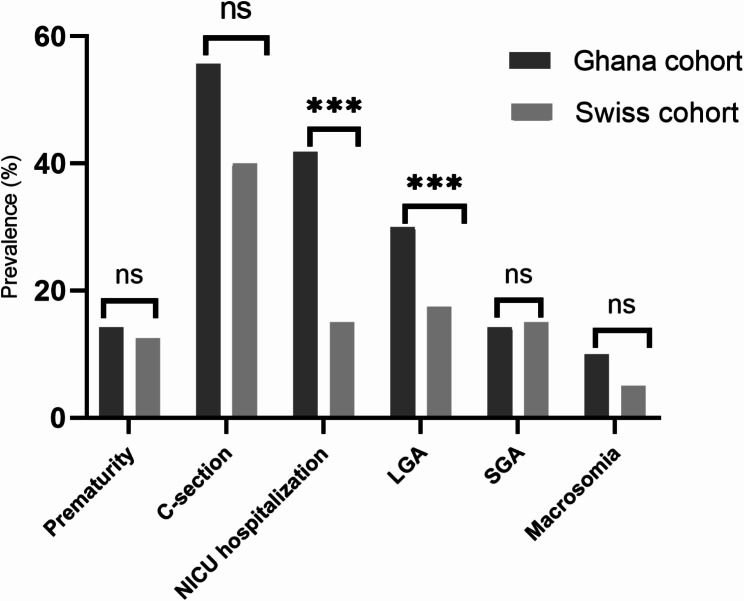
Obstetric and neonatal outcomes in both cohorts. Compared to the Swiss cohort, Women in the Ghana cohort had sinficantly higher rates of C-section, LGA, SGA, and NICU hospitalizations (** denotes *p* < 0.04, *** denotes *p* ≤ 0.02)

Table 4 shows the differences in obstetric and neonatal outcomes between the two cohorts. Birth weight, length, and head circumference and their respective z-scores as well as rate of macrosomia were all similar in both cohorts. Gestational age at delivery and the rates of prematurity as well as neonatal complications were also similar. Both the 1-minute and 5-minute Apgar scores were higher in the SC (both *p* ≤ 0.001), whereas the prevalence of LGA (30% vs. 17%, *p* = 0.031), and the rate of NICU hospitalizations (42% vs. 15%, *p* = 0.041) were higher in the GC (Fig. 2), both independent of HbA1c at GDM diagnosis and of pre-pregnancy BMI. Although the rate of caesarean delivery was higher in the GC (data not shown), this was not independent of HbA1c at GDM booking and pre-pregnancy BMI.

**Table 4:** Adjusted differences in obstetric and neonatal outcomes in women diagnosed with gestational diabetes in Ghana and Switzerland

Variable	Switzerland (*n* = 40)	Ghana (*n* = 70)	*P*.value#	*P*-value*
	Mean ± SD	Mean ± SD		
Gestational age at delivery (weeks)	38.5 ± 1.7	37.5 ± 2.2	**0.021**	0.076
Neonatal birthweight (kg)	3.13 ± 0.61	3.18 ± 0.7	0.687	0.161
Neonatal birthweight z-scores	0.08 ± 1.0	0.53 ± 1.2	0.064	0.529
Birth length (cm)	48.78 ± 2.81	49.11 ± 3.9	0.643	0.219
Birth length z-scores	0.12 ± 1.3	0.61 ± 1.7	0.140	0.696
Head circumference (cm)	34.2 ± 1.4	34.3 ± 2.1	0.813	0.211
Head circumference z-scores	0.74 ± 1.0	0.95 ± 1.5	0.473	0.504
Neonatal Apgar score at 1 min	8.0 ± 2.1	6.6 ± 1.4	**0.001**	**0.013**
Neonatal Apgar score at 5 min	9.2 ± 1.2	8.0 ± 1.1	**< 0.001**	**0.003**
Neonatal sex (n, %)				
Female	18 (45.0)	29 (41.4)	0.841	0.974
Male	22 (55.0)	41 (58.6)		
Macrosomia (yes), (n, %)^1^	2 (5.0)	7 (10.00)	0.483	0.440
Prematurity (yes), (n, %)^2^	5 (12.5)	10 (14.29)	0.793	0.510
Caesarean delivery (yes), (n, %)	16 (40.0)	39 (55.7)	**0.043**	0.154
Stillbirth (yes), (n, %)	0 (0)	1 (1.4)	0.702	0.194
Neonatal complication (yes), (n, %)	7 (17.5)	10 (14.93)	0.329	0.268
NICU hospitalization (yes), (n, %)^3^	6 (15.0)	28 (41.79)	**0.026**	**0.041**
LGA (yes), (n, %)	7 (17.5)	21 (30.0)	**0.003**	**0.035**
SGA (yes), (n, %)	6 (15.0)	10 (14.2)	0.991	0.183

All values are expressed as mean and standard deviation unless otherwise stated

^1^Macrosomia defined as birthweight ≥ 4000 g

^2^Prematurity defined at infant delivery < 37 gestational age

^3^Criteria for neonatal hospitalization differ in Ghana and Switzerland

^#^Unadjsuted *p* values

* *P* values were adjusted for HbA1c at GDM diagnosis and for pre-pregnancy BMI

*P* values derived from Chi-square test was used for categorical variables and ANOVA (ANCOVA for adjusted results) for continuous variables. Bold *p* values are significant (*p* < 0.05)

## Discussion

In this first comparative analysis of metabolic and mental health outcomes of women with GDM in Ghana and Switzerland, two countries with similar GDM prevalence. Women in the GC had 9.1 weeks earlier booking at the GDM clinic, higher pre-pregnancy BMI, lower total GWG, and similar weight at the first GDM visit compared to women in the SC. At GDM diagnosis, metabolic outcomes including fasting glucose, 2 h glucose, HbA1c, and the prevalence of previously undiagnosed diabetes before 15 weeks GA were substantially increased in the GC compared to the SC. Women in the GC had higher well-being scores, but similar depression symptoms. Regarding eating behavior, the SC had a higher score of reliance on hunger and satiety (RHSC) subscale of the IES-2, whereas the GC had increased eating for physical rather than emotional reasons (EPR) subscale scores. Compared to the SC, the rates of LGA, and NICU hospitalizations were higher in the GC independent of HbA1c and pre-pregnancy BMI.

We observed that the GC had a more adverse metabolic profile before and during pregnancy compared to the SC, which was characterized by higher pre-pregnancy BMI and a mean of 8 kg higher pre-pregnancy weight, albeit self-reported, as well as a worsened glucose control and an increased rate of diabetes diagnosed in pregnancy (despite being diagnosed 9 weeks earlier). In term of evolution of weight, the GC had a mean of 5.1 kg lower total GWG, lower rate of weight gain per week during pregnancy but a similar rate of weight gain since GDM diagnosis which is what one would expect in this higher BMI population. Family history of diabetes and personal history of GDM could influence higher glucose control, but these were not more prevalent in the GC, although the lack of a systematic screening of both diabetes and GDM could also have impacted on this prevalence. The differences in health systems, a lower diabetes screening that may have diagnosed pre-existing diabetes earlier and the lack of structured GDM follow-up and management might contribute to the adverse metabolic outcomes observed in the GC. Most striking was the high prevalence of previously undiagnosed T2DM, which was 12% when including all women and 40% when including only women diagnosed < 15 weeks of GA in the GC [1]. This reveals the importance of screening these high-risk women before conception. Regarding glucose-lowering medical treatment for GDM during pregnancy, 84% of the GC received metformin, whereas only 1% of the SC received metformin, due to differences in the health system functioning in both countries, as well as in pricing. A previous study showing that women with GDM treated with metformin had less GWG compared with those treated with insulin could explain some of our results [37]. However, birth weight, prevalence of LGA, and rates of cesarean deliveries are generally reported to be lower with metformin treatment [38, 39]. Therefore, the difference in pre-pregnancy BMI and glucose control prior to and possibly after GDM diagnosis could play a predominant role. A recent study of adherence to GDM testing appointment in Ghana showed that over 40% of pregnant women do not return for the GDM test [40], due to the inefficiency of the health system and lack of education on GDM and its metabolic consequences for both the mother and the child.

The GC had almost 25% higher well-being scores during pregnancy, despite similar symptoms of depression and prevalence of probable diagnosis of depression ((EPDS ≥ 11) in both cohorts. It is possible that the high partner and social support observed in both cohorts (98% vs. 94%) might account for the lack of differences in symptoms of depression. Research indicates that networks of strong social support including partner and family social support provide a protective effect against pregnancy complications including depression [41]. Despite this, general well-being scores in the GC were significantly higher than the SC. The increased level of family social support during pregnancy and communal living in Ghana might explain the higher well-being scores in the GC [42], although this requires further investigation.

Regarding intuitive eating behavior, the GC had an increased EPR score, whereas RHSC was higher in the SC. This suggest that the GC relied more on physical than emotional reasons to eat than the SC, whereas the GC relied less on their hunger and satiety cues to eat compared to SC. For the latter, people in Switzerland might have been sensibilized more, even outside the healthcare setting. The reliance on physical rather than emotional reasons to eat in the GC might be related to the higher well-being score compared to the SC. When the analysis was restricted to the GC only, we found that the EPR subscale was positively associated with well-being, but these findings warrant further investigations.

Obstetric and neonatal outcomes were similar in both cohorts, except that the GC had increased rates of NICU hospitalizations, and LGA. This might be related to differences in healthcare procedures (NICU hospitalizations), but also to differences in glucose control. It is not clear why 42% of neonates in the GC were hospitalized at the NICU but this might be related to the high rate of LGA and the lower APGAR scores, as well as differences in health care systems. Our results are contrary to a study in Ghana that showed that women with GDM had increased adverse birth outcomes including cesarean delivery compared to women without GDM, but this study did not adjust for BMI [18]. In addition, our study did not compare outcomes with women without GDM. In previous studies we reported a prematurity rate of 8-10.8% in our Swiss population which also includes an RCT study [1–3]. The 12% prevalence reported in this study could be due to the low sample size or higher rate of complications. The rate of caesarean delivery in the Ghana cohort might be explained by the fact that this population includes 35% of women with undiagnosed diabetes before 15 weeks GA. This is why we adjusted for BMI and HbA1c in our analyses and these differences were not significant after adjustment.

This study has several strengths. To our knowledge, this is the first study to investigate metabolic and mental health outcomes during pregnancy in women with GDM in Ghana. It is the first to compare all relevant outcomes of metabolic and mental health during pregnancy in women with GDM to Switzerland, a western country with a structured healthcare system with broader universal access to healthcare. Despite this, there are several limitations that need to be highlighted. Whereas Switzerland has a well-defined routine GDM diagnosis, follow-up, and treatment, this was not the case in Ghana. However, women with GDM were diagnosed according to the same international guidelines regarding the cut-offs in both groups. Nevertheless, there were also some substantial differences as GDM was diagnosed much earlier in the GC and women were treated with metformin but less followed with no active accent on lifestyle changes. We also included HbA1c in our diabetes diagnosis before 15 weeks of gestational age which may be controversial. The differences in the health systems in both countries might account for the observed differences between the cohorts. In addition, as this study was a prospective observational study, we did not perform a priori sample size estimation, and thus we may be underpowered to detect differences in some primary outcomes. Other limitations, such as a relatively small sample size, limit our ability to generalize our findings. For the GC, we relied on self-reported pre-pregnancy weight, which may be a limitation. In addition, we have no data on glucose intolerance in early postpartum. Data on hypoglycemia and respiratory distress were not included in the analysis as there were mostly missing in the GC. The inability to include the UPE subscale because of the diet counselling with a dietician is a source of limitation as the overall IES-2 score would have been interesting. Despite these limitations, this study provides useful data on metabolic and mental health outcomes of GDM in Ghana.

## Conclusions

In this first ever comparative analysis of metabolic and mental health outcomes of GDM in Ghana and Switzerland, we observed that despite a mean of 5.1 kg lower total GWG and 5.6 kg lower GWG up to GDM diagnosis, women in the GC had poor glucose control and a higher prevalence of newly diagnosed diabetes before 15 weeks GA compared to the SC. This is probably related to differences in pre-pregnancy BMI and undetected pre-existing diabetes. Although well-being was substantially higher in the GC cohort, symptoms of depression were similar in both groups. In the context of more pre-existing adverse metabolic health, women in the GC had increased rates of LGA, and NICU hospitalization. Further research is needed to investigate the long-term impacts of metabolic and mental health outcomes of GDM in Ghana and the impact of those findings on future generations. This will provide data to develop GDM-specific guidelines to improve pre-conception care as well as diagnosis and management in Ghana.

## Data Availability

The datasets generated and/or analyzed during the current study are not publicly available but are available from the corresponding author on reasonable request.

## Abbreviations

GDM: Gestational diabetes mellitus
GWG: Gestational weight gain
SC: Swiss cohort
GC: Ghana cohort
IADPSG: International association of diabetic pregnancy study groups
OGTT: Oral glucose tolerance test
NAM: National academy of medicine
IOM: Institute of medicine
FPG: Fasting plasma glucose
ANC: Antenatal clinic
ADA: American diabetes association
EPDS: Edinburgh postnatal depression scale
WHO-5: World health organization well-being index
EPR: Eating for physical rather than emotional reasons
RHSC: Reliance on hunger and satiety cues
GA: Gestational age
LGA: Large-for-gestational-age
SGA: Small-for-gestational-age
NICU: Neonatal intensive care unit
BMI: Body mass index
